# Inter-group trade-off between intentions and outcomes in children’s forgiveness

**DOI:** 10.3389/fpsyg.2025.1720622

**Published:** 2025-12-11

**Authors:** Qin Yang, Lu Wang, Yongqin Zhang, Zhengmei Ma, Xiulan Cheng

**Affiliations:** 1 Faculty of Education, Shaanxi Normal University, Xi’an, China; 2 College of Educational Sciences, Changji University, Changji, China; 3 Urumqi Hongqi Kindergarten, Urumqi, China

**Keywords:** children, forgiveness, intention, outcome, inter-group relation, moral judgment, collectivism

## Abstract

**Introduction:**

Forgiveness plays a vital role in establishing and repairing interpersonal relationships, serving as an indispensable component in the harmonious functioning of human society. In the complex group dynamics, children frequently weigh a transgressor’s intentions against the outcomes of transgressions when deciding whether to forgive.

**Methods:**

Experiment 1 involved 178 children aged 4–6 years (*M* = 5.08, *SD* = 0.82) and examined differences in forgiveness under conditions of varying intentions (unintentional vs. intentional) and outcomes (positive vs. negative). Experiment 2 introduced inter-group relations (in-group vs. out-group) and recruited 195 children (*M* = 4.88, *SD* = 0.80) to investigate how intention and outcome are weighed in forgiveness across different inter-group relations.

**Results:**

The results indicate that: (1) Children are capable of recognizing a transgressor’s intention and show more forgiveness to unintentional transgressors compared to intentional ones; (2) Children show more forgiveness to unintentional transgressors who cause negative outcomes than intentional transgressors who yield positive outcomes; (3) When an in-group member commits a transgression, children tend to consider intentions, whereas when an out-group member transgresses, they focus more on the outcomes.

**Discussion:**

In conclusion, this suggests that forgiveness in children involves the integration of multiple factors, which also paves the way for examining how collectivist cultures shape early social-cognitive development.

## 1 Introduction

Throughout the evolutionary process, cooperation and interdependence formed an essential foundation for individual survival (Tomasello et al., 2012), which necessitates that forgiveness serves a vital function in repairing interpersonal bonds when they are damaged by transgressions (McLaughlin et al., 2025). Distinct from the reconciliation involving mutual participation by both parties (Čehajić-Clancy et al., 2016) or externally motivated compliance (Radley and Dart, 2016), forgiveness refers to an individual’s psychological structure to proactive relinquish negative cognitive, emotional, and behavioral responses toward the wrongdoer after experiencing harm, and even to replace them with positive cognitive, emotional, and behavioral responses (Enright, 1991). Forgiveness is observable from the early childhood, as evidenced by children’s tendencies to forgive transgressors (McLaughlin et al., 2024). The rapid growth of possessive awareness in early childhood often triggers conflicts (Rochat, 2011), not only contributes to the frequent interpersonal conflicts during this developmental stage (Hay et al., 2022), but also heightens children’s sensitivity to others’ social and interpersonal cues when forgiveness (Oostenbroek and Vaish, 2019). Therefore, forgiveness in the period is not only crucial for the development of early social adaptation and emotional regulation skills, but also closely linked to the quality of subsequent interpersonal relationships (Fourie et al., 2020; Toda et al., 2024). Although studies have typically examined intention and outcome as independent factors influencing children’s forgiveness, the messy reality of group interactions rarely presents these factors as neatly isolated. Instead, children must grapple with the central challenge of adjudicating between conflicting intention and outcome cues. Beyond this, does this trade-off differ depending on whether the offender belongs to an in-group or out-group? The study aims to examine how transgression intention and outcome influence children’s forgiveness, as well as the role of inter-group relations in this process, thereby revealing the moderators children consider when granting forgiveness.

### 1.1 Transgressor’s intention and children’s forgiveness

The transgressor’s intention is a crucial clue in children’s forgiveness. An individual’s social judgments (e.g., forgiveness, blame) following an transgression are partly based on their perception of the transgressor’s intention, particularly whether the behavior was intentional or unintentional (D'Esterre et al., 2019). Research has shown that children are more willing to forgive unintentional transgressions (Cheng et al., 2025; Koenig et al., 2019; Nobes et al., 2017). For instance, Amir et al. (2021) found that five-year-old children were harsher toward intentional transgressors than toward unintentional transgressors. Building on this, Waddington et al. (2023) discovered that, unlike 4-year-olds, 5-year-olds were more likely to accept apologies from well-intentioned transgressors. That is to say, previous research has generally suggested that 5-year-olds consider transgressor’s intentions when making social moral judgments (Cushman et al., 2013; Li and Tomasello, 2018). However, McElroy et al. (2023) indicated that forgiveness among 5-year-olds is not directly linked to whether the transgression was intentional or unintentional. Therefore, existing research may not yet have reached a consensus on when children can integrate intentional information in forgiveness, and further investigation is needed. Furthermore, while most previous studies have centred on the impact of the transgressor’s apology or compensation on children’s forgiveness (McCullough et al., 2014; Oostenbroek and Vaish, 2019), with few studies directly investigating whether children consider the transgressor’s intention when forgiving. In fact, apologies or compensation may be given under situational pressure and may not fully reflect an individual’s genuine moral standards (McElroy et al., 2023). Therefore, in addition to superficial remedial actions, it is equally crucial to consider the transgressor’s initial intention. Existing research has clearly demonstrated that four-year-old children are capable of making moral judgments based on the transgressor’s intention (Cushman et al., 2013) and that forgiveness can be observed at this age (Cameron et al., 2022; Vaish and Oostenbroek, 2022). This study therefore aims to examine the impact of a transgressor’s intention on forgiveness in children aged 4–6.

### 1.2 The trade-off between intention and outcome in children’s forgiveness

However, real-world decisions about forgiveness are not determined solely by intention. The severity and destructive of the objective outcome resulting from an action also constitute crucial information that children cannot overlook when making forgiveness decisions. Children are often presented with a dilemma in forgiveness when an intentional transgression yields positive outcomes versus an unintentional transgression causing negative consequences, compelling them to integrate and weigh these conflicting cues. Previous research has typically treated intention and outcome as two independent factors influencing children’s forgiveness, and examining how each affects children’s forgiveness separately. For instance, van der Wal et al. (2019) discovered that children make forgiveness decisions based on the severity of the transgression committed by others. Nobes et al. (2017) showed that children are more likely to forgive unintentional transgressors than intentional ones. However, these studies have yet to systematically reveal how children express forgiveness in situations where intentions and outcomes are misaligned. In fact, a key indicator of maturity in children’s moral judgment is the shift from reliance on objective outcomes to an understanding of subjective intentions. According to Kohlberg’s (1981) theory of moral development stages, younger children’s moral judgments are primarily based on the objective outcomes of actions or considerations of self-interest. As they grow older, children gradually begin to place greater emphasis on others’ intentions, social norms, and relational harmony, and become capable of using others’ subjective intention as the central basis for moral judgment. This also partly explains why existing research has found that younger children typically exhibit moral realism, meaning they primarily base moral judgment on the severity of behavioral outcomes. As they grow older, typically around the age of 5 or 6, they begin to move beyond superficial outcomes and integrating the transgressor’s intention as a core basis for moral judgment (Baird and Astington, 2004; Killen et al., 2011; Margoni and Surian, 2020; Proft and Rakoczy, 2019). Although previous studies have explored the trade-off between intentions and outcomes in moral domains such as resource distribution and rule compliance (Van de Vondervoort and Hamlin, 2018; Zhang et al., 2019; Williams and Moore, 2014), how children integrate information about the transgression’s intentions and outcomes in order to forgiveness remain an important issue that has not yet been systematically revealed. As a context that inherently presents moral dilemmas, forgiveness involves conflicts among multiple values such as emotion, rules, and fairness, making it an ideal window through which to observe children’s reasoning patterns and decision-making logic across different stages of moral development. The aim of this study is therefore to explore how young children weigh up intentions and outcomes when it comes to forgiveness.

### 1.3 The moderating role of inter-group relations

More importantly, children’s forgiveness are deeply embedded within their social inter-group relation networks. In other words, the relationship between the victim and the transgressor influences children’s decisions to forgive, meaning victims may not forgive all transgressors equally. Turner et al. (1979) suggested that individuals categorize people into “us” (in-group member) and “them” (out-group member) based on inter-group relations, and generally exhibit in-group favoritism. Previous research has shown that this in-group bias in forgiveness emerges early in development (Oostenbroek and Vaish, 2019). For example, Peets et al. (2013) found that children were show more forgiveness when the transgressor was an in-group rather than an out-group member. Similarly, Vaish and Oostenbroek (2022) observed that 5-year-olds showed greater tolerance toward in-group than out-group transgressors. In summary, these findings suggest that young children’s forgiveness is, to some extent, shaped by in-group and out-group dynamics. The deeply rooted interdependence among humans fosters profound psychological bonds between individuals and groups. As a key socio-psychological mechanism, these bonds shape individuals’ cognition, emotion, and behavior toward different inter-group relations (Fiske, 2012). Their most direct manifestation is the formation of a stable in-group bias, whereby individuals systematically tend to favor, trust, and prioritize members of their in-group over those of out-groups (Otten, 2016).

Interestingly, inter-group relations as a key social-contextual variable, may moderate the impact of intention and outcome on forgiveness. When a transgressor belongs to the in-group, children may be more inclined to attribute benign intentions to their actions or downplay the negative consequences, thereby demonstrating higher levels of forgiveness. In contrast, when the transgressor is from an out-group, the same transgression may be interpreted as stemming from more malicious motives, thus inhibiting forgiveness (Molenberghs and Louis, 2018; Vaish and Oostenbroek, 2022). This suggests that forgiveness in children is not merely an individual moral decision, but also a product of social cognition shaped by group dynamics (McCauley et al., 2022). However, previous studies have often treated inter-group relation as an isolated factor or examined its interaction with either intention or outcome in isolation, without fully integrating the tripartite influence of inter-group relation, intention, and outcome. For instance, Liu et al. (2025) found that children tend to attribute more benign intentions to in-group members, and Geraci et al. (2024) observed that children are more likely to associate negative outcomes with out-group members. Thus, what role does inter-group relation play when intention and outcome interact to shape children’s forgiveness? Does it amplify the dominance of intention, or does it mitigate the severity of the outcome? Research addressing these questions remains limited, to address this gap, the present study aims to investigate how inter-group relation moderates the effects of transgression intention and outcome on forgiveness in young children.

### 1.4 The present study

A significant amount of prior research has shown that children usually observe and evaluate the behavior of transgressors from a bystander perspective (Oostenbroek and Vaish, 2019). While this third-party perspective is important, it does not equate to genuine forgiveness. Forgiveness is fundamentally an internal experience and an active process initiated by the victim, since only the victim has the ability to repair the relationship (Zitek et al., 2010). Furthermore, children’s emotional responses (e.g., sadness, anger) are often less intense when they are unaffected observers than when they are direct victims (Metcalfe and Mischel, 1999). In first-person contexts, these heightened emotional reactions may actually inhibit the formation of forgiveness, consequently slowing its developmental trajectory (Blake et al., 2014). Therefore, this study adopts the victim perspective to examine how transgressor’s intention and outcome influence children’s forgiveness, while also investigating the role of inter-group relations in this process, with the aim of systematically elucidating the factors of children’s forgiveness.

## 2 Experiment 1

### 2.1 Method

#### 2.1.1 Participants

A total of 183 children aged 4–6 years, primarily from Chinese middle-class families, were initially recruited for the study. Due to experimenter error (*n* = 1), inattention (*n* = 2), and study withdrawal (*n* = 2), 5 children were excluded. The final sample consisted of 178 children (46% girls; *M* = 5.08, *SD* = 0.82). Data were collected in June 2025, none of the children had prior experience with similar experiments, and all experimental procedures were conducted with assent from the children and informed consent from their guardians.

#### 2.1.2 Design

A two-factor mixed experimental design was employed, with transgression intention (intentional vs. unintentional) and outcome (positive vs. negative) as independent variables. The dependent variable was children’s forgiveness, operationalized as the number of flowers (0–10) shared with the transgressor. Participants were randomly assigned to one of four conditions: intentional transgression-positive outcome, unintentional transgression-positive outcome, intentional transgression-negative outcome, and unintentional transgression-negative outcome. Each child was tested individually in a quiet room, and the entire behavioral experiment was recorded by camera. Both the experimenter and research assistants conducting the sessions were trained graduate students and teachers.

#### 2.1.3 Materials

The experimental materials consist of paper, paint, two boxes, 10 flowers, a table, two chairs, and a photograph of a neutral expression representing the experimenter.

#### 2.1.4 Procedure

The task was adapted from the experimental paradigm used by Oostenbroek and Vaish (2019), which has been widely used to study forgiveness in 4–6 year olds (Vaish and Oostenbroek, 2022; Vaish and Savell, 2022).

The experimenter guided the child into the testing room. The child and experimenter sat on the same side of a table with blank paper and painting materials placed in front of them, while the moderator sat opposite. The moderator explained to both the child and experimenter: “Thank you all for coming to paint, I will now give each of you a small gift. Everyone may paint freely according to their own ideas. Upon completion of the painting, an additional reward will be allocated based on the creation. However, should the recipient be unable to complete the task, the gift will be returned.” The moderator then temporarily left the room, citing other matters to attend to. Several minutes later, both the child and experimenter successfully completed their painting tasks. The experimenter expressed interest in viewing the child’s artwork, but during the viewing process, stained the child’s painting.

*Unintentional Condition*: The experimenter accidentally spilled paint on the drawing and expressed regret after the mistake, saying, “I’ve stained your painting. I just wanted to take a closer look. I did not mean for this to happen. It’s all my fault.”

*Intentional Condition*: The experimenter deliberately splashes paint on the painting and, began laughing and smirking, emphasizing that the behavior was deliberate, then remarked casually, “Oh, I meant to stain it.”

Upon completion of above procedures, the moderator (who was previously unaware of the specific incident occurring during the experiment) returned to the testing room and instructed the experimenter to organize the materials and leave. The moderator then noticed the damaged painting and gently asked the child, “What happened?” If the child could clearly and completely describe the transgression by stating, “The experimenter stained my painting,” then proceeded to the subsequent questioning phase. If the child provided an incomplete response (e.g., “My painting got dirty”), the moderator would offer prompts such as, “How did it get dirty? Who stained it?” to assist the child in recalling the event fully.

Once the child accurately described the transgression, if they could repeat what the experimenter said after damaging the painting—for example, “She said he did not expect it to happen, it was her fault” or other statements reflecting intention, the moderator would continue with further questions. If the child forgot or could not clearly recall the experimenter’s remarks, the moderator would prompt them to remember the specific expressions used by the experimenter.

These steps ensured that all children, before proceeding to the formal test phase, were fully aware of the incident involving the damaged painting and could accurately distinguish whether the experimenter’s transgression was “intentional” or “unintentional.” Then, drawing on previous research (Oostenbroek and Vaish, 2019; Waddington et al., 2023), the following test questions were posed to measure children’s understanding of intention:

1. Do you like the experimenter?
2. Do you dislike the experimenter?
3. If you needed a crayon, do you think experimenter would help you get one?
4. If you drew another picture, do you think experimenter would help you color it carefully?
5. If you drew another picture, do you think experimenter would take good care of it?

All questions are mandatory, and each item is presented sequentially in a predetermined order. When a child provides an ambiguous response, the moderator will guide them to attempt a specific selection. If the child offers no response, the moderator will repeat the question. Should the child remain unresponsive after the repeated question, the procedure will advance to the next item in the testing sequence.

Upon seeing the damaged painting, the host responds differently based on the experimental condition.

*Positive Outcome*: “Your painting actually looks more beautiful with these added strokes. Congratulations on completing the task so well. I’m going to give you an extra reward.”

*Negative Outcome*: “Your painting has been stained. Unfortunately, you did not successfully complete the task. According to the rules, I need to take back the reward you received earlier.”

This phase was completed independently by the child under the guidance of the moderator. The moderator gave the child 10 flowers and explained: “Here are 10 flowers. The experimenter will come back to check her box. If you’d like, you may take some of these 10 flowers and put them in her box.” The moderator then temporarily turned away to attend to other matters. If the child inquired about how to distribute the flowers, the moderator would encourage them to decide according to their own wishes. After the children complete the distribution, the moderator will further inquire about their reasoning, such as: “Why did you want to give her these flowers?” This helps confirm whether the children understood the task rather than merely following instructions, children may respond freely. It is important to note that the flower distribution task serves as a reliable measure of children’s forgiveness, it effectively captures children’s sensitivity to moral judgments about others’ motives and has been widely used in forgiveness research among children (McElroy et al., 2023; Oostenbroek and Vaish, 2019).

Following the experiment, the experimenter returned to the room with the host and provided the child participant with an age-appropriate explanation of the study’s true purpose, accompanied by a sincere apology. Subsequently, the experimenter collaborated with the child to create a new painting and presented the child with a small toy as a token of appreciation for their participation in the experiment.

### 2.2 Results

**Figure 1:**
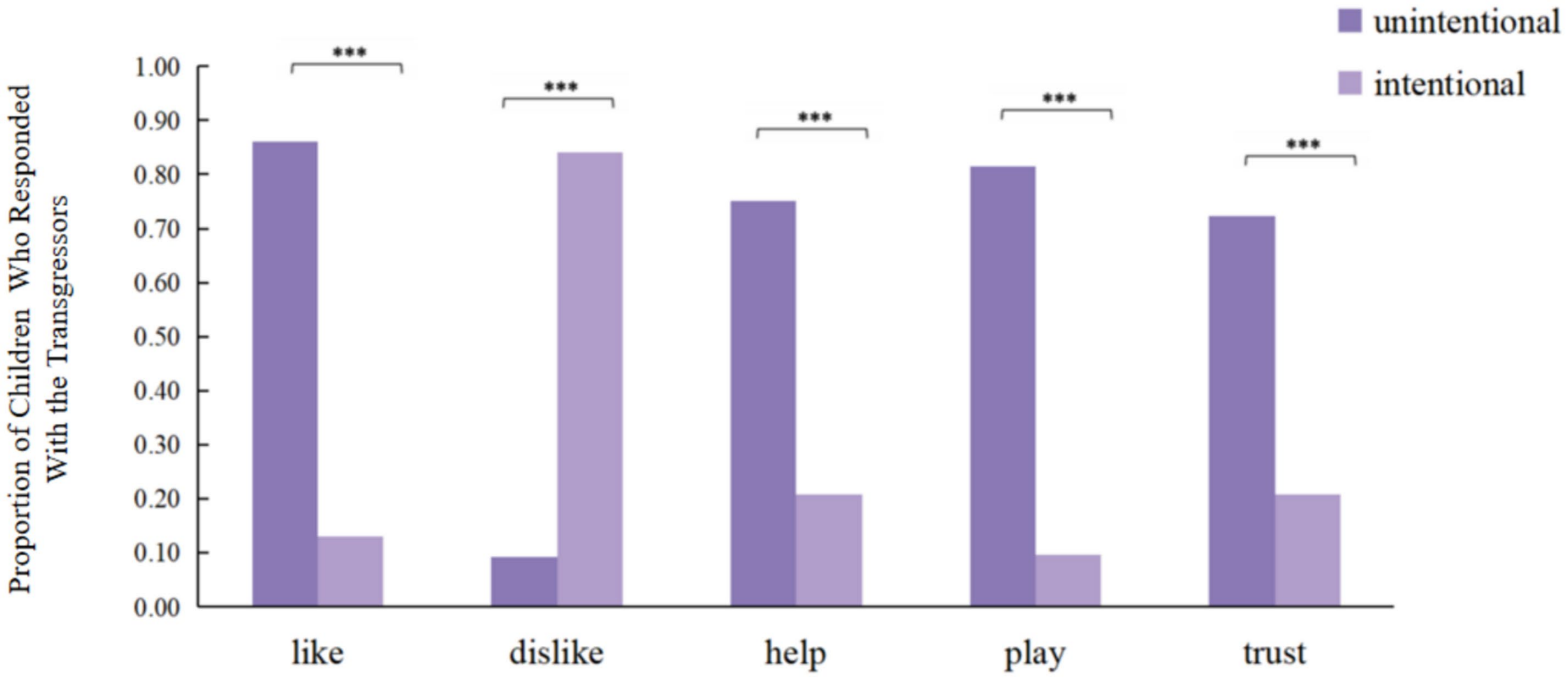
Proportion of children in Experiment 1 who responded with the transgressors. *p < 0.05, **p < 0.01, ***p < 0.001.

As shown in Figure 1, the children’s response proportions for the five questions regarding unintentional and intentional transgressors are presented. The results of chi-square tests of independence revealed statistically significant associations between intention (unintentional vs. intentional) and children’s responses across all questions (*p* < 0.001). This indicates that children systematically distinguished between behaviors based on intention and made differential moral judgments toward the transgressors accordingly, confirming that the experimental scenario successfully elicited children’s consideration of intention. Furthermore, chi-square tests showed no significant differences in this intention understanding ability by children’s gender or age (*p* > 0.05), suggesting that children as young as 4 years old were able to comprehend the transgressor’s intention.

A two-way repeated measures *ANOVA* was conducted with intention (intentional, unintentional) and outcome (positive, negative) as independent variables and children’s forgiveness (number of flowers shared with the transgressor, 0–10) as the dependent variable and the results showed:

A significant main effect of transgression intention, *F*(1,174) = 151.563, *p* < 0.001, *η_p_*^2^ = 0.466, with children showed more forgiveness for unintentional (*M* = 7.91, *SD* = 1.36) than intentional transgressors (*M* = 5.18, *SD* = 1.91).

A significant main effect of transgression outcome, *F*(1,174) = 39.546, *p* < 0.001, *η_p_*^2^ = 0.695, with children showed more forgiveness toward transgressors who led to positive outcomes (*M* = 7.25, *SD* = 1.97) compared to those who led to negative outcomes (*M* = 5.86, *SD* = 2.09).

**Figure 2:**
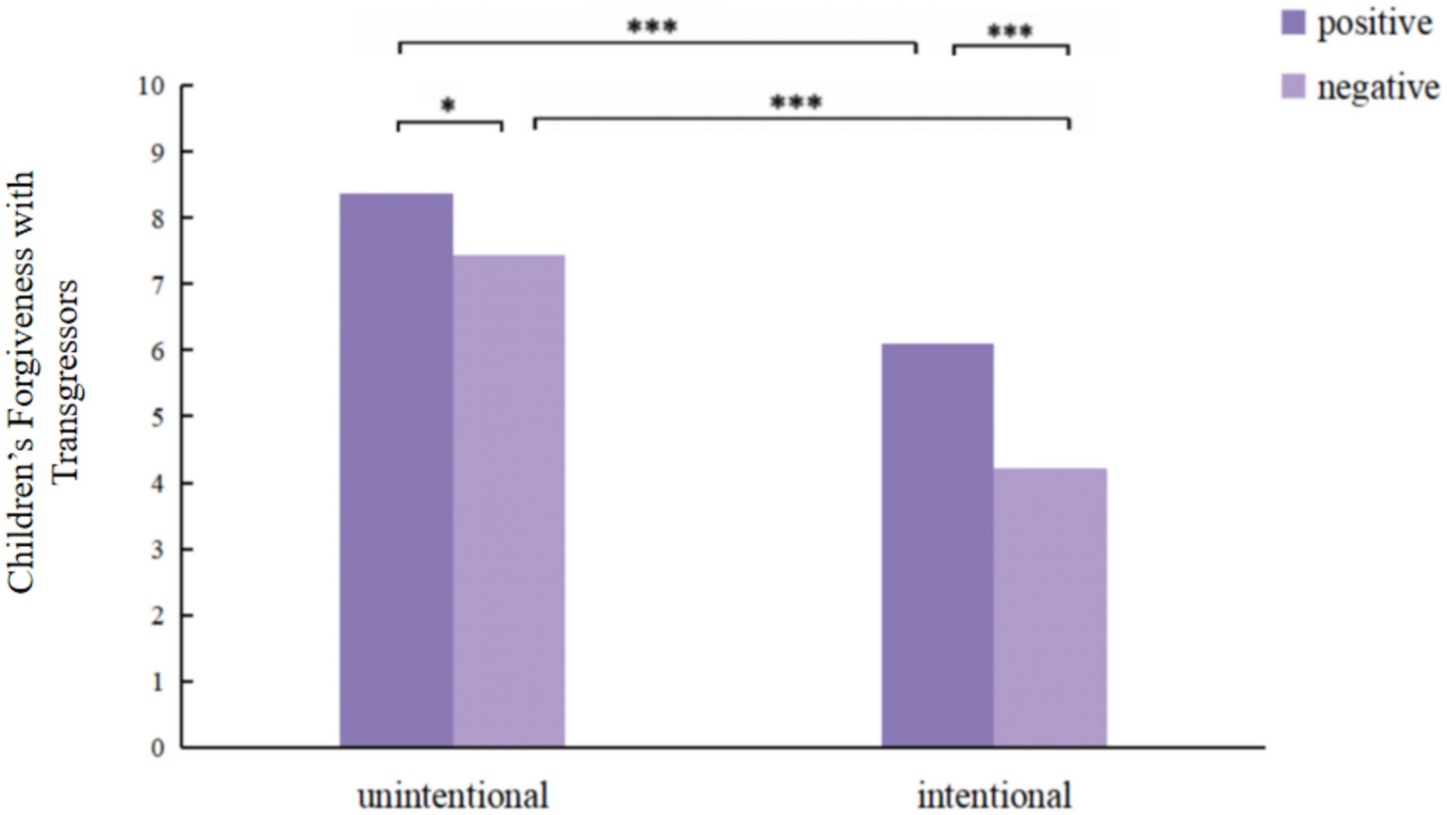
Children’s forgiveness (out of 10) with transgressors on different intention and outcome. *p < 0.05, **p < 0.01, ***p < 0.001.

A significant interaction was observed between transgression intention and outcome, indicating that children’s forgiveness was jointly influenced by both factors, *F*(1,174) = 4.487, *p* < 0.05, *η_p_*^2^ = 0.025. Simple effects analysis (Figure 2) revealed that children consistently showed more forgiveness toward transgressors associated with positive outcomes regardless of intention, specifically manifested as, in unintentional transgression conditions, children’s forgiveness was significantly higher for positive outcomes (*M* = 8.36, *SD* = 1.11) compared to negative outcomes (*M* = 7.43, *SD* = 1.45), *F*(1, 174) = 8.896, *p* < 0.05, *η_p_*^2^ = 0.049; in intentional transgression conditions, children’s forgiveness was significantly higher for positive outcomes (*M* = 6.09, *SD* = 2.01) compared to negative outcomes (*M* = 4.21, *SD* = 1.20), *F*(1, 174) = 34.560, *p* < 0.001, *η_p_*^2^ = 0.166. Similarly, children consistently showed more forgiveness toward unintentional transgressors across outcome conditions: for positive outcomes, children showed more forgiveness toward unintentional transgressors (*M* = 8.36, *SD* = 1.11) than toward intentional transgressors (*M* = 6.09, *SD* = 2.01), *F*(1, 174) = 53.760, *p* < 0.001, *η_p_*^2^ = 0.236; for negative outcomes, children showed more forgiveness toward unintentional transgressors (*M* = 7.43, *SD* = 1.45) than toward intentional transgressors (*M* = 4.21, *SD* = 1.20), *F*(1, 174) = 100.707, *p* < 0.001, *η_p_*^2^ = 0.367. Notably, follow-up comparisons revealed that children showed more forgiveness to unintentional transgressors with negative outcomes (*M* = 7.43, *SD* = 1.45) than intentional transgressors with positive outcomes (*M* = 6.09, *SD* = 2.01), *t*(87) = 4.633, *p* < 0.05.

### 2.3 Discussion

The results indicate that children were generally able to discern the transgressor’s intention and showed more forgiveness to unintentional transgressors. This finding is partially consistent with previous research (Waddington et al., 2023; McElroy et al., 2023). Consistently, children showed more forgiveness once they understood the transgressor’s were unintentional. In contrast, McElroy et al. (2023), employing the same research paradigm as this study, found that Western 4-year-olds could not genuinely comprehend the intentions expressed by transgressors and showed no difference in forgiveness between unintentional and intentional transgressors, the 4-year-olds in this Chinese sample accurately recognized the transgressor’s intention. Furthermore, children showed more forgiveness to unintentional transgressors who caused negative outcome than intentional transgressors who brought about positive outcome. This finding is consistent with previous research, which suggests that children’s ability and propensity to employ intentionality in moral reasoning grows during the preschool years (Chernyak and Sobel, 2016; Li and Tomasello, 2018; Killen et al., 2011). This finding lends further support to the notion of a positive correlation between the intention of transgressors and the forgiveness of victims among Chinese children. Overall, it is posited that collectivist cultures may exert a distinct influence on children’s social cognitive development (e.g., theory of mind, perspective taking) in comparison to individualist cultures.

## 3 Experiment 2

### 3.1 Method

#### 3.1.1 Participants

A total of 204 children aged 4–6 years, primarily from Chinese middle-class families, were initially recruited for the study. Due to experimenter error (*n* = 3), inattention (*n* = 1), emotional distress (*n* = 3), or study withdrawal (*n* = 2), 9 children were excluded. The final sample consisted of 195 children (44% girls; *M* = 4.88, *SD* = 0.80). Data were collected in June 2025, none of the participants had taken part in similar previous studies or overlapped with participants from Experiment 1. All procedures were conducted with assent from the children and informed consent from their guardians.

#### 3.1.2 Design

Experiment 2 builds upon Experiment 1 by introducing inter-group relations, employing a three-factor mixed design, with between-subjects independent variables comprising transgression intention (intentional, unintentional) and outcome (positive, negative), and within-subjects independent variables comprising inter-group relations (ingroup, outgroup). The dependent variable in this study was the forgiveness exhibited by the children in question, measured by the number of flowers they shared with their transgressor. Participants were randomly assigned to one of four conditions same as Experiment 1. It is noteworthy that the present study utilised a forced-choice paradigm, in which subjects were presented with members of both the ingroup and the outgroup concurrently, and were required to select one member from each group. In contrast to single-subject scenarios, this contrastive condition has been shown to more effectively stimulate children’s discernment abilities (Boseovski and Lee, 2006; Ronfard and Lane, 2018; Vaish and Oostenbroek, 2022). Consequently, it enables a more efficient examination of their capacity to integrate group-related factors into forgiveness.

#### 3.1.3 Materials

Same as Experiment 1.

#### 3.1.4 Procedure

The experimental and operational procedures employed in this study are analogous to those utilised in Experiment 1. The key modification involved randomly assigning child and experimenters to distinct groups. Child participants and in-group experimenter A wore matching yellow hats and wristbands, while out-group experimenters wore green hats and wristbands (Vaish and Oostenbroek, 2022). Following this, in-group member A accompanied the child in cooperative play with shared objectives as a warm-up activity to enhance in-group identification. After the warm-up concluded, the facilitator asked the children which group they belonged to. Upon receiving accurate responses, Experimenters A and B then led the children into the testing room to formally commence the experiment. The child was seated at one end of the table, with both experimenters positioned on either side of the child. To control for potential confounding effects of experimenter characteristics, the two experimenters alternated roles as the in-group transgressor (Experimenter A) and out-group transgressor (Experimenter B) across participants. Furthermore, to mitigate order effects, we implemented systematic counterbalancing of both experimenter seating positions and the sequence of option A or B presentation in test questions across all participants.

### 3.2 Results

**Figure 3:**
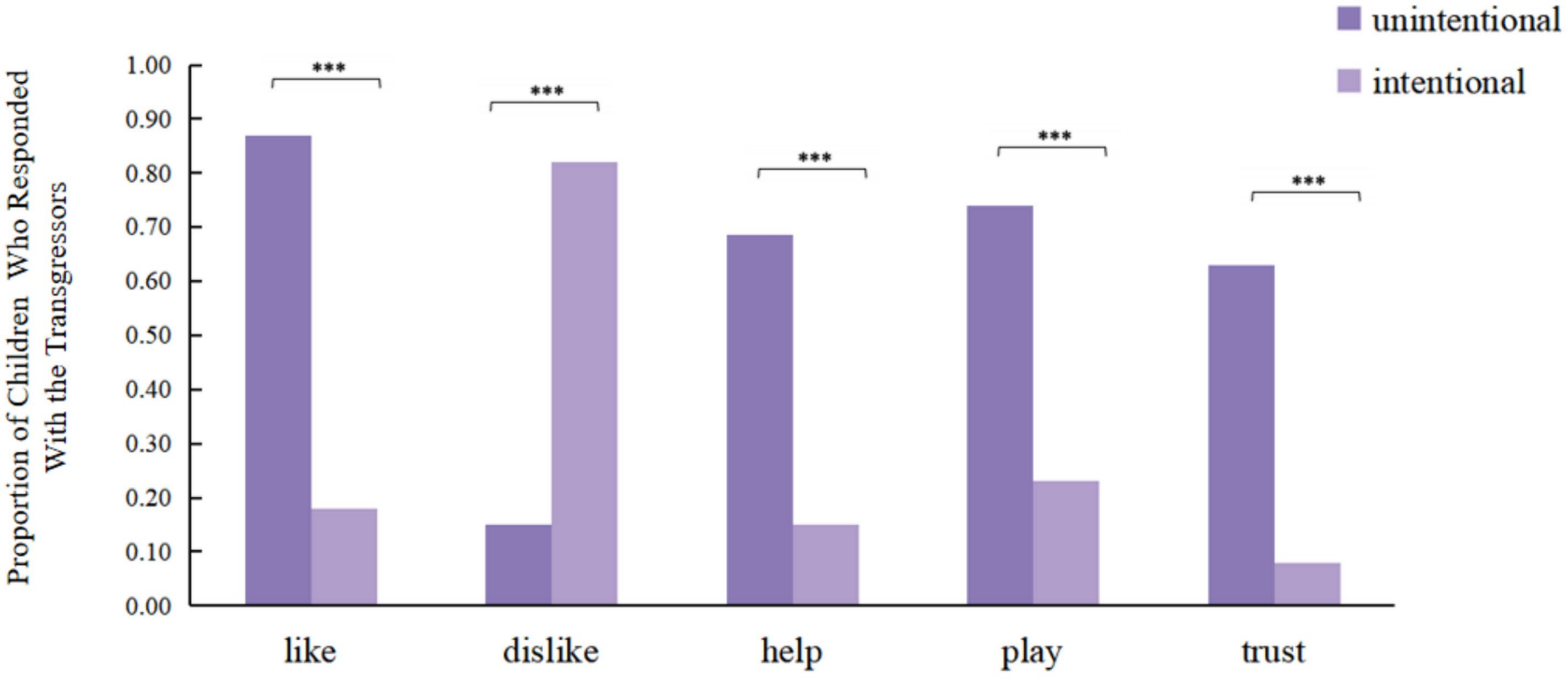
Proportion of children in Experiment 2 who responded with the transgressors. *p < 0.05, **p < 0.01, ***p < 0.001.

Experiment 2 validated the findings of Experiment 1 regarding children’s understanding of intention. A highly significant association was observed between intention (unintentional vs. intentional) and children’s responses across all five questions (*p* < 0.001) (Figure 3). Furthermore, chi-square test results indicated no significant differences in this intention comprehension ability with respect to children’s gender or age (*p* > 0.05), suggesting that children are already capable of understanding the intentions of transgressors.

A multi-factorial repeated-measures *ANOVA* was conducted with intention, outcome, and inter-group relation as independent variables and children’s forgiveness as the dependent variable. The results revealed.

A significant main effect of transgression intention, *F*(1,191) = 135.916, *p* < 0.001, *η_p_*^2^ = 0*.*416, with children showed more forgiveness for unintentional (*M* = 7.78, *SD* = 2.01) compared to intentional transgressors (*M* = 5.56, *SD* = 2.01).

A significant main effect of transgression outcome, *F*(1,191) = 96.059, *p* < 0.001, *η_p_*^2^ = 0.335, with children showed more forgiveness toward transgressors who led to positive outcomes (*M* = 7.60, *SD* = 2.01) compared to those who led to negative outcomes (*M* = 5.74, *SD* = 2.01).

A significant main effect of inter-group relation, *F*(1, 191) = 41.857, *p* < 0.001, *η_p_*^2^ = 0.180, with children showed more forgiveness toward in-group transgressors (*M* = 7.06, *SD* = 2.01) compared to out-group transgressors (*M* = 6.28, *SD* = 2.01).

The interaction between transgression outcome and inter-group relation was not statistically significant, *F*(1,191) = 2.691, *p* > 0.05, *η_p_*^2^ = 0.014.

**Figure 4:**
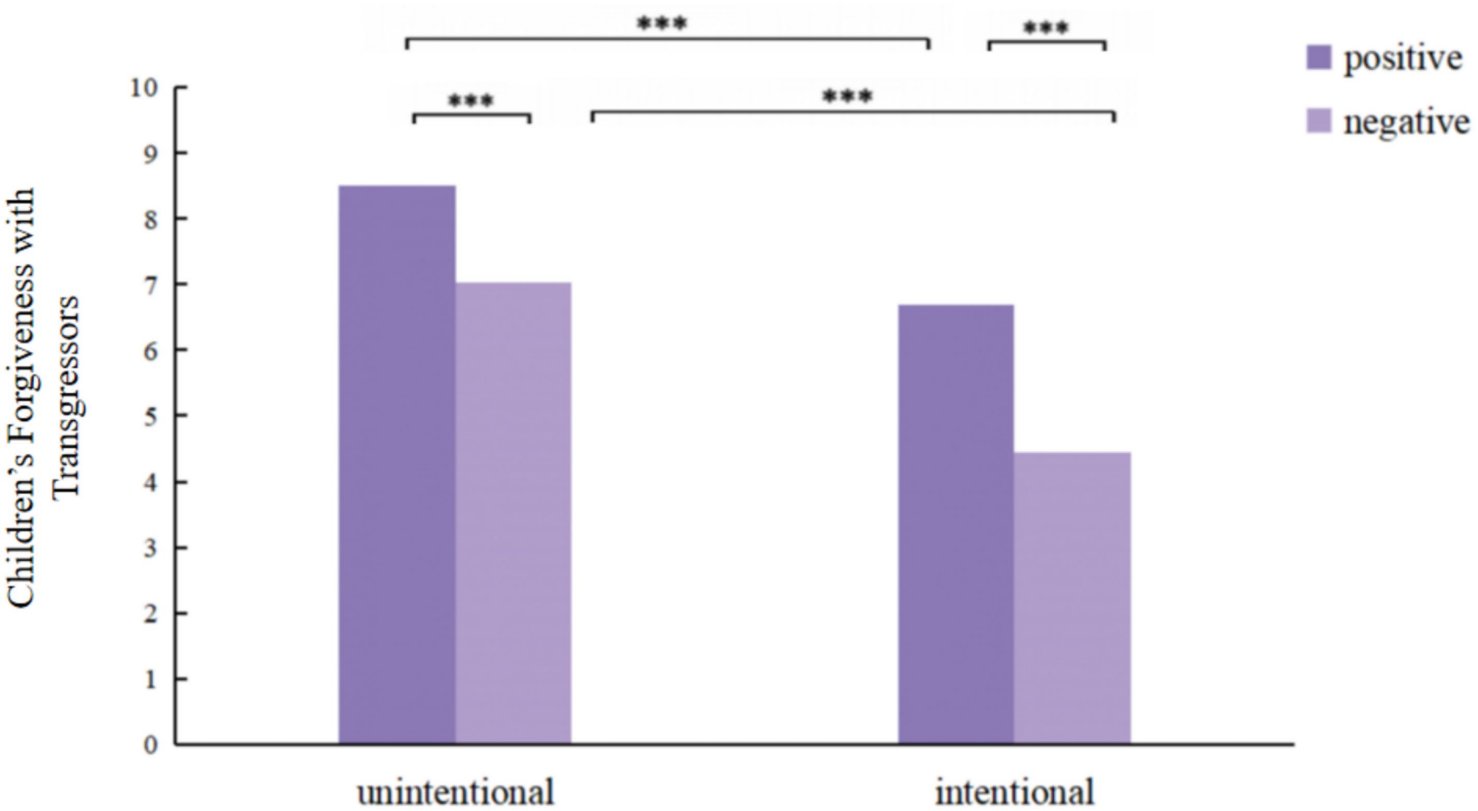
Children’s forgiveness (out of 10) with transgressors on different intention and outcome. *p < 0.05, **p < 0.01, ***p < 0.001.

A significant interaction was observed between transgression intention and outcome, indicating that children’s forgiveness was jointly influenced by both factors, *F*(1,191) = 4.221, *p* < 0.05, *η_p_*^2^ = 0.022. Simple effects analysis (Figure 4) revealed that children consistently showed more forgiveness toward transgressors associated with positive outcomes regardless of intention, specifically manifested as, in unintentional transgression conditions, children’s forgiveness was significantly higher for positive outcomes (*M* = 8.51, *SD* = 2.64) compared to negative outcomes(*M* = 7.04, *SD* = 2.58), *F*(1, 191) = 30.472, *p* < 0.001, *η_p_*^2^ = 0.138; in intentional transgression conditions, children’s forgiveness was significantly higher for positive outcomes (*M* = 6.68, *SD* = 2.67) compared to negative outcomes (*M* = 4.44, *SD* = 2.66), *F*(1, 191) = 69.213, *p* < 0.001, *η_p_*^2^ = 0.266. Similarly, children consistently showed more forgiveness toward unintentional transgressors across outcome conditions: for positive outcomes, children showed more forgiveness toward unintentional transgressors (*M* = 8.51, *SD* = 2.64)than toward intentional transgressors (*M* = 6.09, *SD* = 2.01), *F*(1, 191) = 45.889, *p* < 0.001, *η_p_*^2^ = 0.194; for negative outcomes, children showed more forgiveness toward unintentional transgressors (*M* = 7.04, *SD* = 2.58) than toward intentional transgressors (*M* = 4.44, *SD* = 2.66), *F*(1, 191) = 94.489, *p* < 0.001, *η_p_*^2^ = 0.331.

**Figure 5:**
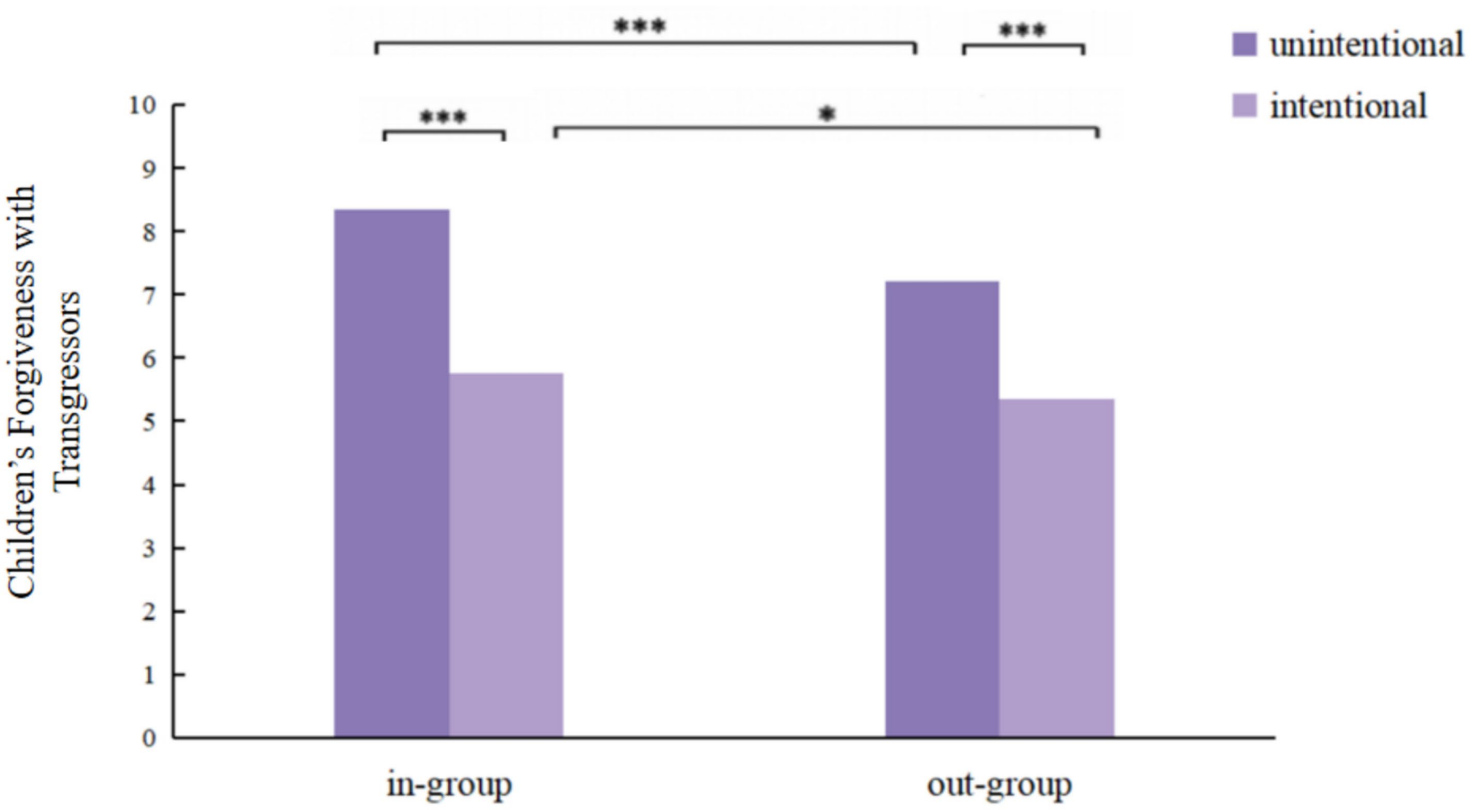
Children’s forgiveness (out of 10) with transgressors on different intention and inter-group relation. *p < 0.05, **p < 0.01, ***p < 0.001.

A significant interaction was observed between transgression intention and inter-group relations, *F*(1,191) = 8.862, *p* < 0.01, *η_p_*^2^ = 0.044. Simple effects analysis (Figure 5) revealed the following patterns, regardless of the transgressor’s group affiliation, children showed a consistent preference for forgiving unintentional transgressors, specifically, for in-group transgressors, children showed more forgiveness toward unintentional transgressors (*M* = 8.34, *SD* = 2.21) compared to intentional transgressors (*M* = 5.77, *SD* = 2.25), *F*(1, 191) = 98.519, *p* < 0.001, *η_p_*^2^ = 0.338; for out-group transgressors, children also showed more forgiveness toward unintentional transgressors (*M* = 7.21, *SD* = 2.18) compared to intentional transgressors (*M* = 5.35, *SD* = 2.22), *F*(1, 191) = 48.088, *p* < 0.001, *η_p_*^2^ = 0.199. Children consistently showed more forgiveness toward in-group transgressors compared to out-group transgressors regardless of intention. Specifically, in unintentional transgression conditions, children showed more forgiveness toward in-group transgressors (*M* = 8.34, *SD* = 2.21) than toward out-group transgressors (*M* = 7.21, *SD* = 2.18), *F*(1, 191) = 44.574, *p* < 0.001, *η_p_*^2^ = 0.188; in intentional transgression conditions, children also showed more forgiveness toward in-group transgressors (*M* = 5.77, *SD* = 2.25) than toward out-group transgressors (*M* = 5.35, *SD* = 2.22), *F*(1, 191) = 5.863, *p* < 0.05, *η_p_*^2^ = 0.029.

**Figure 6:**
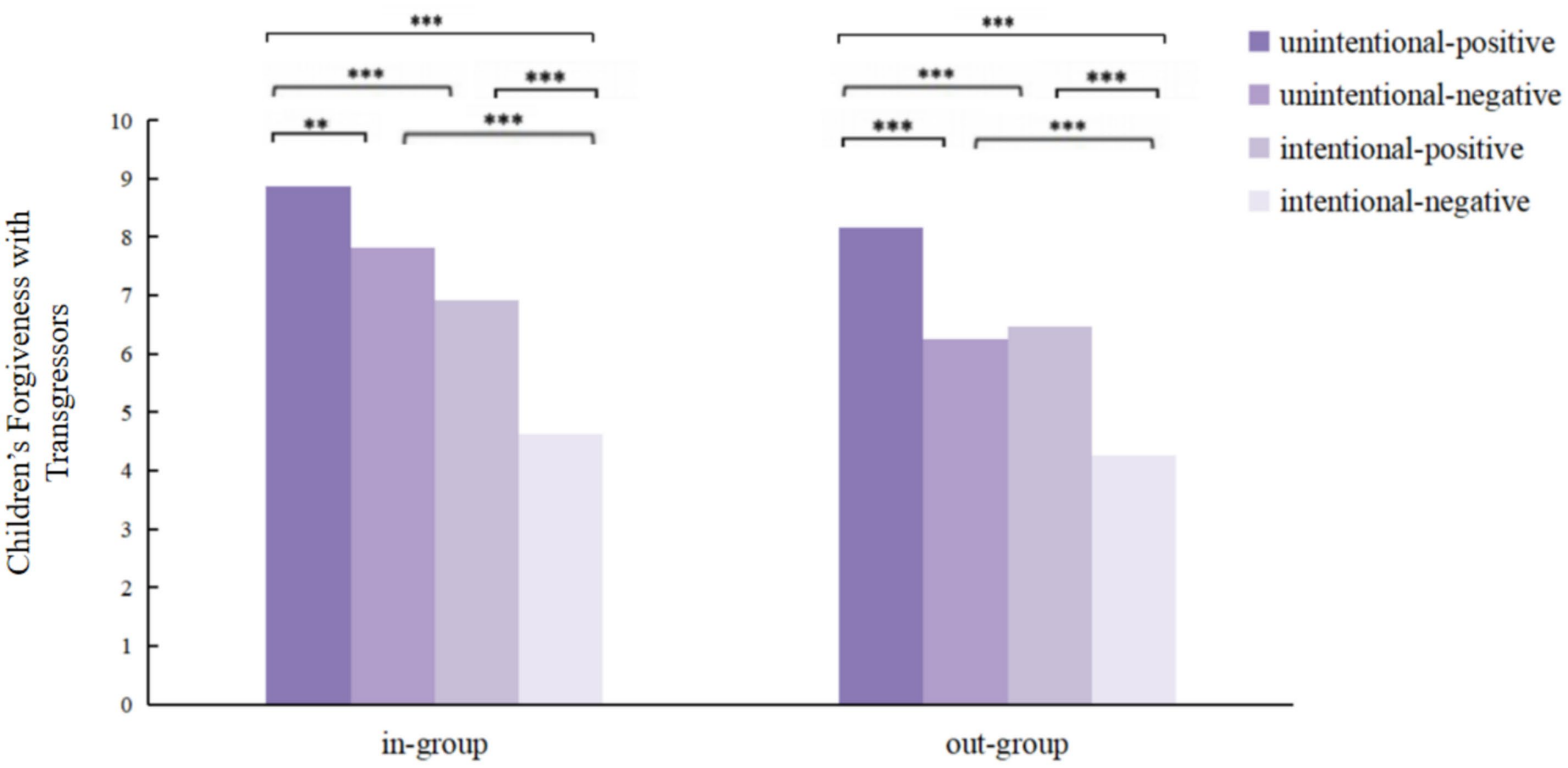
Children’s forgiveness (out of 10) with transgressors on different intention, outcome, and inter-group relation. *p < 0.05, **p < 0.01, ***p < 0.001.

A significant three-way interaction was observed among transgression intention, outcome, and inter-group relations, *F*(1,191) = 3.959, *p* < 0.05, *η_p_*^2^ = 0.020, indicating that the interaction pattern between intention and outcome was moderated by group affiliation. The results of the simple effect analysis (Figure 6) show that: when the transgressors are in-group members, in unintentional transgression conditions, children showed more forgiveness toward those with positive outcomes (*M* = 8.86, *SD* = 0.89) compared to those with negative outcomes (*M* = 7.82, *SD* = 1.44), *F*(1, 191) = 10.720, *p* < 0.01, *η_p_*^2^ = 0.053; in intentional transgression conditions, children showed more forgiveness toward those with positive outcomes (*M* = 6.92, *SD* = 1.99) compared to those with negative outcomes (*M* = 4.63, *SD* = 1.77), *F*(1, 191) = 50.757, *p* < 0.001, *η_p_*^2^ = 0.210; in positive outcome conditions, children showed more forgiveness toward unintentional transgressors (*M* = 8.86, *SD* = 0.89) compared to intentional transgressors (*M* = 6.92, *SD* = 1.99), *F*(1, 191) = 36.768, *p* < 0.001, *η_p_*^2^ = 0.161, in negative outcome conditions, children also showed more forgiveness toward unintentional transgressors (*M* = 7.82, *SD* = 1.44) compared to intentional transgressors (*M* = 4.63, *SD* = 1.77), F(1, 191) = 100.672, *p* < 0.001, *η_p_*^2^ = 0.345. Further analysis revealed that when the transgressor belonged to the in-group, children were significantly more forgiving of unintentional transgressors with negative outcomes (*M* = 7.82, *SD* = 1.44) than intentional transgressors with positive outcomes (*M* = 6.92, *SD* = 1.99), *t* (94) = 2.598, *p* < 0.05. When the transgressors are out-group members, in unintentional transgression conditions, children showed more forgiveness toward those with positive outcomes (*M* = 8.16, *SD* = 1.25) compared to those with negative outcomes (*M* = 6.26, *SD* = 1.29), *F*(1, 191) = 37.123, *p* < 0.001, *η_p_*^2^ = 0.163; in intentional transgression conditions, children showed more forgiveness toward those with positive outcomes (*M* = 6.46, *SD* = 1.92) compared to those with negative outcomes (*M* = 4.25, *SD* = 1.67), *F*(1, 191) = 48.468, *p* < 0.001, *η_p_*^2^ = 0.202; in positive outcome conditions, children showed more forgiveness toward unintentional transgressors(*M* = 8.16, *SD* = 1.25) compared to intentional transgressors(*M* = 6.46, *SD* = 1.92), *F*(1, 191) = 29.187, *p* < 0.001, *η_p_*^2^ = 0.133; in negative outcome conditions, children also showed more forgiveness toward unintentional transgressors (*M* = 6.26, *SD* = 1.29) compared to intentional transgressors (*M* = 4.25, *SD* = 1.67), *F*(1, 191) = 40.973, *p* < 0.001, *η_p_*^2^ = 0.177. Further analysis revealed that when the transgressor belonged to the in-group, children were significantly more forgiving of intentional transgressors with positive outcomes (*M* = 6.46, *SD* = 1.92) than unintentional transgressors with negative outcomes (*M* = 6.26, *SD* = 1.29), *t*(94) = − 0.457, *p* < 0.05.

### 3.3 Discussion

Building on the findings of Experiment 1, Experiment 2 founded that children’s forgiveness are not fixed but are significantly influenced by inter-group relations. Specifically, when the transgressor was an in-group member, children showed a greater tendency to attribute weight to intention; conversely, when the transgressor was from an out-group, they relied more heavily on outcome. This pattern reflects an in-group bias in children’s forgiveness, which aligns with existing literature. For instance, Vaish and Oostenbroek (2022) found that even under equivalent conditions, 5-year-olds exhibited greater leniency toward in-group transgressors, demonstrating early emerging in-group favoritism. This phenomenon may be attributed to the collectivist cultural context in which young children are situated. In cultures emphasizing collectivism, children are oriented toward maintaining social harmony and group cohesion. As a result, they often adopt strategies such as conflict avoidance, suppression of personal emotions (e.g., anger), and internal attribution to preserve relational stability and continuity within the group (Wu et al., 2018). It follows, then, that forgiveness and reconciliation are frequently employed as vital means of restoring social relationships (Ho and Worthington, 2020; Martínez–Lozano et al., 2011). This cultural framework leads children to adopt an intention-oriented forgiveness strategy toward in-group members, while applying a stricter, outcome-oriented standard toward out-group members, reflecting a culturally embedded cognition of differential treatment. Thus, collectivist culture appears to shape not only the content of moral judgments but also the early development of differentiated cognitive strategies in moral evaluation.

## 4 General discussion

### 4.1 Transgressor’s intention and children’s forgiveness

Experiment 1 revealed a significant correlation between transgressor’s intentions and children’s forgiveness, Experiment 2 further validated this finding, consistent with previous studies involving Western children (Chernyak and Sobel, 2016; McElroy et al., 2023; Waddington et al., 2023), indicating that the role of intent in forgiveness judgments exhibits cross-cultural consistency. In the context of interpersonal conflict contexts, assessing whether a transgression was committed intentionally and granting forgiveness accordingly serves as a foundational component for repairing relationships and sustaining positive interaction or cooperation (Struthers et al., 2008). The consistency of this ability across children from diverse sociocultural backgrounds indicates that the development of this interpersonal strategy occurs during the early stages of socialization, around the age of five (Rochat et al., 2009; Vaish and Savell, 2022; Wu et al., 2018). Notably, compared to previous studies using similar experimental paradigms, the study further revealed that even 4-year-olds founded the capacity to discern the intentions of transgressor and show more forgiveness toward those who act unintentionally. This represents a substantial developmental difference within a short period, potentially indicating meaningful cultural variations in social-cognitive development (Kadiangandu et al., 2007). Within collectivist cultural contexts, social norms have been found to encourage and reward altruistic behavior (Rochat et al., 2009), which may prompt earlier emergence of altruistic tendencies (Wu and Su, 2014) and greater emphasis on suppressing personal emotions or actions to maintain interpersonal harmony (Günsoy et al., 2015). In summary, this study indicates that the age at which young children accurately infer others’ transgression intention may be as early as 4-years-old. Given the notable divergence from prior findings, further replication is needed, particularly with careful consideration of variables such as parental education and socioeconomic status. Moreover, it remains unclear whether this capacity emerges even earlier, and whether more culturally adapted research paradigms could detect relevant abilities in even younger children.

### 4.2 The trade-off between intention and outcome in children’s forgiveness

It has been found that children are show more forgiveness to transgressors who yield positive outcomes than those who yield negative ones. This finding is consistent with prior research suggesting that young children possess the capacity to make judgments based on outcomes (van der Wal et al., 2019). For instance, Riek and DeWit (2018) discovered that children were more likely to forgive transgressors whose actions resulted in less severe outcomes. However, this outcome-oriented judgment capacity may represent an early form of moral decision-making in young children (Buon and Margoni, 2025). This study further reveals that even when outcomes are positive, children’s forgiveness is not unconditional, intentions influences the weight assigned to outcomes in moral judgment. Specifically, even when the outcome is positive, intentional transgressions still fails to earn forgiveness, with the degree of forgiveness remaining notably low; when the transgressor acts unintentionally, the moral weight of the outcome diminishes considerably. Although negative outcomes still receive less forgiveness than positive ones, the gap in forgiveness between the two is far narrower compared to scenarios involving intentional transgressions. This finding is consistent with the conclusions of a seminal study by Vanderbilt et al. (2023), which found that young children exhibited a greater propensity to share stickers with a benevolent yet less generous partner as opposed to a selfish yet generous one. This may be because children at this stage have already developed a certain degree of sensitivity to others’ intentions. When cognitive resources are limited, intention information tends to take precedence in moral judgment (Waddington et al., 2023). Even if the outcome is positive, children still perceive intentional transgressions as a red flag in moral evaluation. Furthermore, children are not only capable of perceiving intentions but can also use intention and outcome information to infer the reliability of the transgressors (Nobes et al., 2009). Intentional transgressions, even without causing actual harm, are regarded as a violation of social contracts, which undermines the foundation of interpersonal trust. Conversely, unintentional transgressions, even when leading to negative outcomes, are understood as excusable accidents. Overall, this finding suggests that children’s moral judgment system is a multi-layered and complex framework. They can not only rely on the most apparent and easily processed outcome information to make quick judgments but have also preliminarily developed the ability to integrate deeper social information such as intentions, demonstrating the flexibility and depth of early moral cognition.

These findings also indicate that during early childhood, children gradually rely less on outcomes and increasingly attend to the intentions of the transgressor when making moral judgments (Waddington et al., 2023). That is, the tendency to use intentional information in moral reasoning increases throughout the preschool years (Chernyak and Sobel, 2016; Li and Tomasello, 2018). However, a substantial corpus of earlier research argued that young children primarily base their judgments on outcomes (Baird and Astington, 2004; Karniol, 1978; Piaget, 1965; Zelazo et al., 1996). This discrepancy may be attributed to methodological limitations in earlier studies, which may not have fully captured young children’s nuanced capacities. In contrast, more recent investigations which have aided by developmentally sensitive measures and advances in theory of mind research, suggest that the emergence of intention-oriented moral evaluation occurs earlier than previously thought and follows a more complex, gradual developmental trajectory (Nobes et al., 2017). Although evidence supports the universality of this developmental process, its underlying psychological mechanisms and developmental trajectories remain to be clarified. Future research should integrate longitudinal designs and neuroscience methods to further elucidate the internal processes of children’s moral judgment development. Additionally, attention should be paid to the cross-cultural applicability of these findings. Cross-cultural research has revealed that in certain small-scale “opacity of mind” societies (e.g., Fiji), individuals place greater emphasis on outcomes rather than intentions when evaluating transgressions (McNamara et al., 2019). Consequently, more cross-cultural studies are needed to delve into the mechanisms underlying children’s moral judgments based on intentions versus consequences and their cultural specificity.

### 4.3 Intergroup trade-off between intentions and outcomes in children’s forgiveness

Inter-group relations moderate children’s forgiveness, specifically manifesting as a greater tendency to consider intentions when in-group members commit transgressions, while focusing more on outcomes when out-group members err. This discrepancy may stem from the group identity and sense of belonging gradually developed during early socialization. Research indicates that inter-group relations influence children’s moral judgments to a certain extent (Baron and Dunham, 2015; Liberman et al., 2018), and that moral judgment serves as a prerequisite and foundation for forgiveness decisions (Koenig et al., 2019). One key reason for the in-group bias observed in young children’s social behavior relates to the implicit moral attributions they assign to in-group members (Zhang et al., 2021). For instance, when evaluating others’ adherence to social norms, children establish varying psychological distances, which subsequently shape moral judgments (Aguilar et al., 2013; Amit and Greene, 2012; Chalik and Dunham, 2025; Körner and Volk, 2014; Sudo and Ishikawa, 2025) and lead to different forgiveness decisions. Specifically, young children exhibit a forgiveness strategy that places greater emphasis on intention toward in-group members because they more readily feel empathy and psychological closeness with them, making them more willing to deeply understand the motivations behind their actions. Even when outcomes are negative, good intentions may still be forgiven (Farooq et al., 2022; Mulvey et al., 2020; Yuly–Youngblood et al., 2022). In contrast, when dealing with out-group members, children often lack trust and emotional connection, exhibiting a tendency toward “de-mentalization” (Fiske, 2012), making them more prone to adopt a cautious and defensive attitude (Arini et al., 2025; Glidden et al., 2021; Mulvey et al., 2022). As a result, they focus more on the actual consequences of the behavior rather than the intent behind it. Furthermore, this finding aligns with the profound influence of collectivist culture, which advocates for conflict resolution that emphasize harmony and adheres to the principle of distinction between in-group and out-group. This cultural framework provides a plausible explanation for the observed behavior (Ho and Fung, 2011; Paz et al., 2008). In short, children’s early social cognitive schemas are shaped by the collectiveist cultural context through multiple channels, family parenting practices that emphasise interpersonal harmony and cultivate sharing and cooperation skills, kindergarten education that prioritizes collective interests and teamwork, prevailing sociocultural narratives that promote in-group solidarity and out-group vigilance. Together, these influences enable children to apply different moral standards when managing relationships with in-groups and out-groups, thus fostering their active participation in cultural practices.

In summary, children’s forgiveness already showed considerable complexity, that not simply “outcome-oriented” or “intention-oriented” in their reasoning. Rather, they flexibly adjust their judgment criteria based on whether an actor belongs to their in-group. Thus, this study not only reveals group bias in children’s forgiveness decisions but also indicates at a deeper level that moral development does not occur in a vacuum. Instead, it is shaped by the sociocultural environment through daily practices. Cultural logic begins to be internalized as early as childhood, guiding moral decision-making and subtly molding cognitive tendencies in handling social conflicts. By the preschool years, children are already learning to integrate basic social, psychological, and behavioral information to make flexible social and moral judgments. Inter-group relations serve as a key contextual variable, modulating the weight given to intentions versus outcomes in children’s moral judgments. However, the underlying mechanisms driving this preference remain to be fully explored. For instance, does this bias stem more from positive favoritism toward the in-group or from negative bias against the out-group? Future research could help distinguish between these explanations by introducing more neutral baseline conditions or by measuring children’s emotional responses. Furthermore, exploring how educational interventions might guide children to overcome this bias and develop more inclusive forgiveness concepts represents a direction with significant practical value.

### 4.4 Limitations and prospects

This study has certain inherent limitations due to constraints in the research conditions. Based on these limitations, it proposes some future research directions. First, the experimental scenarios, though using common transgressions, differ from real-life contexts and may not fully capture the complexity of forgiveness. For instance, due to ethical concerns associated with simulating harm inflicted by acquaintances on children, the critical variable of offender identity (stranger or friend) was not considered Future studies should adopt more ecologically valid paradigms to observe forgiveness in natural settings. Second, current measures rely heavily on behavioral observations, which are subjective and fail to reveal internal processes. Neuroimaging studies with adults show heightened brain activity related to forgiveness (Ohtsubo et al., 2018, 2020). Future research could use brain imaging or eye-tracking to objectively examine children’s cognitive mechanisms in intention-outcome processing. Finally, the short research period limited observations to immediate responses. Longitudinal studies are needed to explore how transgressor characteristics influence forgiveness developmentally and interact with other factors. Overall, despite these limitations, this study contributes empirical evidence to understanding the boundary conditions and underlying factors of forgiveness in young children. It not only extends and deepens the foundation of research on early social cognition but also provides a basis for systematically fostering the quality development of social skills in children.

## 5 Conclusion

This study offers new insights into how transgressor characteristics influence children’s forgiveness. The results revealed: (1) Children show more forgiveness towards transgressors who commit unintentional transgressions or whose actions have a positive outcome. (2) Children are more inclined to forgive those who unintentionally cause negative outcomes than transgressors who intentionally cause positive ones. (3) Children’s forgiveness were critically shaped by group affiliation, characterized by a primary reliance on intention for in-group transgressors and a significant shift toward outcome-based evaluation for out-group transgressors. These findings indicate that young children’s forgiveness decisions are not based on isolated factors, but rather involve weighing and integrating multiple considerations—including the transgressor’s internal motivation, external outcomes, and their own inter-group relationship with the transgressor. These also offers a valuable framework for examining children’s interpersonal forgiveness through the lens of group dynamics, thereby enriching our understanding of how social cognition develops within a complex interplay of characteristics.

## Data Availability

The raw data supporting the conclusions of this article will be made available by the authors, without undue reservation.
